# An evaluation of UV protection imparted by cotton fabrics dyed with natural colorants

**DOI:** 10.1186/1471-5945-4-15

**Published:** 2004-10-27

**Authors:** Ajoy K Sarkar

**Affiliations:** 1 Design and Merchandising, Colorado State University, Fort Collins, Colorado, USA

## Abstract

**Background:**

The ultraviolet properties of textiles dyed with synthetic dyes have been widely reported in literature. However, no study has investigated the ultraviolet properties of natural fabrics dyed with natural colorants. This study reports the Ultraviolet Protection Factor (UPF) of cotton fabrics dyed with colorants of plant and insect origins.

**Methods:**

Three cotton fabrics were dyed with three natural colorants. Fabrics were characterized with respect to fabric construction, weight, thickness and thread count. Influence of fabric characteristics on Ultraviolet Protection Factor was studied. Role of colorant concentration on the ultraviolet protection factor was examined via color strength analysis.

**Results:**

A positive correlation was observed between the weight of the fabric and their UPF values. Similarly, thicker fabrics offered more protection from ultraviolet rays. Thread count appears to negatively correlate with UPF. Dyeing with natural colorants dramatically increased the protective abilities of all three fabric constructions. Additionally, within the same fabric type UPF values increased with higher depths of shade.

**Conclusion:**

Dyeing cotton fabrics with natural colorants increases the ultraviolet protective abilities of the fabrics and can be considered as an effective protection against ultraviolet rays. The UPF is further enhanced with colorant of dark hues and with high concentration of the colorant in the fabric.

## Background

High, short-term exposure to ultraviolet radiation (UVR) from the sun causes sunburns and long-term exposure leads to skin cancer. The National Toxicology Program, U.S. Department of Health and Human Services has classified UVR as a known human carcinogen [1]. The American Cancer Society estimates that more than one million cases of skin cancer cases are diagnosed each year in the United States [2]. In 2002, an estimated 54,200 new cases of melanoma skin cancer alone were diagnosed [2]. A primary reason for the increased incidence of skin cancers is attributed to ozone depletion. Each one percent decrease in ozone concentration is predicted to increase the rate of skin cancer by two percent to five percent [3]. The United States Environmental Protection Agency estimates that ozone depletion will lead to between three and fifteen million new cases of skin cancer in the United States by the year 2075. Other reasons for the skin cancer epidemic can be traced to lifestyle changes such as excessive exposure to sunlight during leisure activities, for example, playing outdoors and swimming in the case of children and golfing and fishing in the case of adults. In the case of agricultural and other outdoor workers, exposure to the sun is an occupational hazard as they have no choice about the duration of their exposure to the sun [3-5].

The ultraviolet radiation (UVR) band consists of three regions: UV-A (320 to 400 nm), UV-B (290 to 320 nm), and UV-C (200 to 290 nm). UV-C is totally absorbed by the atmosphere and does not reach the earth. UV-A causes little visible reaction on the skin but has been shown to decrease the immunological response of skin cells [3]. UV-B is most responsible for the development of skin cancers [3].

Other than drastically reducing exposure to the sun, the most frequently recommended form of UV protection is the use of sunscreens, hats, and proper selection of clothing. Unfortunately, one cannot hold up a textile material to sunlight and determine how susceptible a textile is to UV rays. Even textiles which seem to be non-light transmitting may pass significant amounts of erythema-inducing UV irradiation [4]. Therefore, knowledge of the factors that contribute to the protective abilities of textiles is vital. Important factors include fiber composition, fabric construction and wet-processing history of the fabric such as color and other finishing chemicals that may have been applied to the textile material.

To the author's knowledge, no study has investigated the ultraviolet properties of natural fabrics dyed with natural colorants. A plethora of previous studies have concluded that good UVR protection can be provided by synthetic fibers dyed with high concentrations of synthetic dyes. However, synthetic fibers such as polyester are hydrophobic and are generally not deemed to be comfortable for wear especially in warm weather. According to a report in America's Textile Industries [6] natural fibers are back in demand. The emergence and popularity of a more natural way of life as reflected in a return to organic farming and natural foods has now extended into textiles where the resurgence of natural fibers and natural dyes is on the increase [6,7]. It is hoped that data from the present study will be useful for dermatologists advising patients regarding the UV-protective properties of clothing made from natural fibers and dyed with natural colorants.

In this study, cotton fabrics were dyed with three natural colorants of plant and insect origin. Fabrics were characterized with respect to fabric construction, weight, thickness and thread count. Ultraviolet Protection Factor (UPF) was measured using a labsphere^® ^UV-100 F Ultraviolet Transmission Analyzer. The effect of colorant concentration on the ultraviolet protection factor was examined via color strength analysis using a HunterLab ColorQuest XE^® ^spectrophotometer.

## Methods

Three fabrics were chosen to cover the gamut of basic weave constructions. They were a bleached, mercerized plain weave cotton fabric, a bleached mercerized cotton twill and a desized and bleached cotton sateen. Fabric weight was measured according to ASTM Test Method D3776-96 [8]. Fabric thickness was measured according to ASTM Test Method D1777-96 [8]. Thread counts were measured according to ASTM D3775-98 [8].

Natural plant colorants used were madder (*Rubia tinctorum*) and indigo (*Indigofera tinctoria*) and the natural colorant of insect origin was cochineal (*Dactylopius coccus*). Since natural dyes do not have affinity for cellulosic fibers an alum mordant was used to impart affinity. Fabrics were mordanted prior to dyeing by treating with alum at boil for 45 minutes. The liquor ratio was 1:40 and alum concentration was 10% on weight of the fabric. After mordanting, fabric was squeezed thoroughly and dyed.

Madder and cochineal dyeings were done in stainless steel canisters of an Atlas launder-ometer using 2%, 4% and 6% dye on weight of fabric. The liquor-goods ratio was 40:1. Fabrics were introduced into the dyeing solutions at room temperature. Temperature was raised to the boil and dyeing continued at the boil for 60 minutes. After dyeing, fabrics were rinsed in deionized water, washed using a non-ionic detergent and air-dried. Three replications were done for each colorant and at each dye concentration.

Dyeing with indigo was done in the following manner. Indigo dye was made into a paste and solubilized using sodium hydroxide and sodium hydrosulfite. Fabrics were introduced into dyebaths containing 2%, 4% and 6% dye on weight of fabric. The liquor-goods ratio was 40:1. After thirty minutes of dyeing the fabrics were removed and oxidized by drying in air. The fabrics were then rinsed in deionized water and washed using a non-ionic detergent and dried.

Direct and diffuse UV transmittance through a fabric is the crucial factor determining the UV protection of textiles [9]. Ultraviolet protection factor (UPF) is the scientific term used to indicate the amount of Ultraviolet (UV) protection provided to skin by fabric. UPF values are analogous to SPF values the only distinction being that SPF values for sunscreens are determined through human testing whereas UPF values are based on instrumental measurements [10]. UPF is defined as the ratio of the average effective UV irradiance calculated for unprotected skin to the average UV irradiance calculated for skin protected by the test fabric [5,10]. The higher the value, the longer a person can stay in the sun until the area of skin under the fabric becomes red [5,10]. An effective UVR dose (ED) for unprotected skin is calculated by convolving the incident solar spectral power distribution with the relative spectral effectiveness function and summing over the wavelength range 290-400 nm. The calculation is repeated with the spectral transmission of the fabric as an additional weighting to get the effective dose (ED_m_) for the skin when it is protected. The UPF is defined as the ratio of ED to ED_m _and calculated as follows [11]:



where:

E_*λ *_= erythemal spectral effectiveness

S_*λ *_= solar spectral irradiance in Wm^-2^nm^-1^

T_*λ *_= spectral transmittance of fabric

Δ_*λ *_= the bandwidth in nm

*λ *= the wavelength in nm

UPF's were measured *in vitro *using a labsphere^® ^UV-100 F Ultraviolet Transmission Analyzer according to standard AS/NZ 4399:1996 [12]. Fabrics with a UPF value in the range 15 – 24 were classified as having "Good UV Protection"; when the UPF values were between 25 and 39 fabrics were classified as having "Very Good UV Protection" and "Excellent UV Protection" classification was used when the UPF was 40 or greater [13]. In no event was a fabric assigned a UPF rating greater than 50.

Measured UPF values were also correlated to the color strength of the dyed fabrics. Color strength was evaluated using K/S values generated by a HunterLab ColorQuest XE diffuse/8° spectrophotometer. K/S is a function of color depth and is represented by the equation of Kubelka and Munk (Equation 2). Higher the value of K/S greater is the color strength [14,15].



where R is the reflectance of the dyed fabric; K is the sorption coefficient, and S is the scattering coefficient. The spectrophotometer was standardized for a 1 inch diameter specimen viewing aperture in reflectance – specular included mode. Illuminant D65 and CIE 10-degree observer were used. During measurements, fabric samples were held flat and securely using a spring-loaded sample clamp. Three measurements were taken on each dyed fabric with the fabric rotated between measurements.

## Results and discussion

Fabric characterization parameters and UPF values prior to dyeing are listed in Table 1. Based on the classification parameters referenced previously the plain weave fabric and the sateen weave fabric cannot be rated as offering any degree of protection since their UPF values were less than 15. The undyed twill weave fabric with a UPF of 19.2 is rated as having Good UV Protection. The UPF values of the undyed fabrics can be explained in terms of fiber composition and fabric construction. In terms of fiber composition it is known that undyed bleached cotton, linen, acetate, and rayon fabrics afford poor protection against UV radiation [16]. Fabric construction parameters of weight and thickness show a positive correlation with UPF values. Higher the weight and thicker the fabric, higher is the degree of protection afforded by the fabric. Accordingly, the twill weave fabric with a weight of 235 g/m^2 ^and a thickness of 0.069 centimeters has the highest UPF value followed by the sateen weave fabric which weighed 235 g/m^2 ^and was 0.061 centimeters thick. The plain weave fabric with a weight of 120 g/m^2 ^and a thickness of 0.035 centimeters offers no protection against transmittance of UV rays. The positive correlation between fabric weight and fabric thickness with UPF values can be explained with reference to porosity. Porosity is a measure of tightness of weave and is also called as Coverfactor. Cover factor is defined as the percentage area occupied by warp and filling yarns in a given fabric area [4,17]. The closer the weave, the more is the percentage area occupied by the yarns and more opaque is the fabric to UV radiation. Cover factor is increased by an increase in weight per unit area. Heavier fabric minimizes UV transmission by virtue of having smaller spaces between yarns thus blocking more radiation [3,17]. A related variable is thickness. Thicker, denser fabrics transmit less UV radiation and have a higher cover factor [10]. The data also reveals a negative correlation between thread count and UPF. Higher the thread count, lower is the degree of protection afforded by the fabric. The plain weave fabric with a thread count of 205 had a UPF of 3.2 whereas the twill weave fabric with a thread count of 81 had a UPF of 19.2 with the sateen weave between the two with a thread count of 106 and a UPF of 13.3. A possible explanation for the negative correlation between thread count and UPF is the fact that fabrics that are thinner tend to contain finer yarns and therefore have the highest thread counts [10]. In other words thickness and thread count are inversely correlated a point substantiated by the values in Table 1.

**Table 1:** Fabric characterization parameters and % UV transmittance of undyed fabrics

	Weight, g/m^2^	Thickness, cm.	Thread Count (per inch)	UPF	UV Protection Class
Plain weave	120	0.035	205	3.8	No Class
Twill weave	258	0.069	81	19.2	Good
Sateen weave	235	0.061	106	13.3	No Class

**Figure 1:**
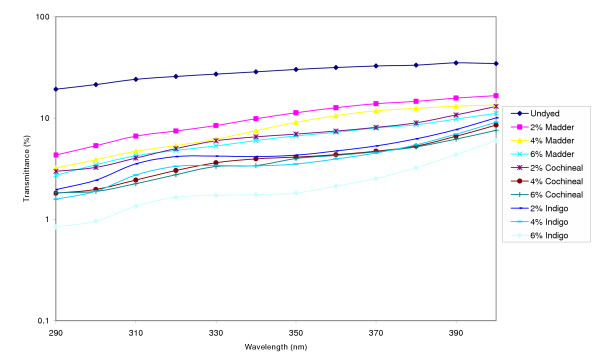
UV transmission of plain weave fabric in the absence and presence of colorants.

The percent UV transmittance data in the presence and absence of colorants for the plain weave fabric is shown in Figure 1. It is noted that since the relative erythemal spectral effectiveness is higher in the UV-B region compared to the UV-A region, the UPF values depend primarily on transmission in the UV-B region. Undyed plain weave fabric had significant transmittance and consequently a very low UPF value of 3.8. UPF values and protection categories of the plain weave fabric dyed with the different colorants are listed in Table 2. As is evident from the transmission data and the corresponding UPF values all colorants used in the study caused a dramatic reduction in UV radiation transmission through the plain weave fabric. The increase in UPF values in the presence of colorant was especially significant for the cochineal and indigo dyed samples which were classified as having Very Good (UPF values between 25 and 39) to Excellent UV Protection (UPF values 40 or greater). Madder dyed samples could be classified as having Good UV Protection (UPF values between 15 and 24) to Very Good UV Protection. Compared to cochineal and indigo, madder is a paler color and therefore these results agree with previous data reported by Reinert et al. [18] who showed that pale colored fabrics of cotton, silk, polyamide, and polyamide/elastan gave less protection against intense UV radiation. The results also show that UPF values for colorants applied at higher concentrations gave higher UPF values. For example, the UPF of the plain weave fabric at a 2% indigo on weight of fabric was 43.1 and that increased to greater than UPF 50 at an indigo concentration of 6%. We agree with Gies et al. [11] who indicated that dyeing fabrics in deeper shades and darker colors improves sun protection properties. Thus although the studies by Reinert at al. and Gies et al. were done with synthetic dyes their conclusions seem to hold with natural colorants as well.

**Table 2:** UPF values, protection class and K/S values of plain weave fabric dyed with natural colorants at different concentrations

	Colorant	UPF	UV Protection Class	K/S
Plain weave	2% Madder	11.1	No Class	0.20
	4% Madder	15.8	Good	0.28
	6% Madder	16.6	Good	0.38
	2% Cochineal	28.5	Very Good	0.63
	4% Cochineal	34	Very Good	0.79
	6% Cochineal	36.6	Very Good	0.99
	2% Indigo	43.1	Excellent	1.78
	4% Indigo	> 50	Excellent	2.56
	6% Indigo	> 50	Excellent	3.02

The K/S values of the dyed fabrics which are a measure of color depth seem to support the claim that higher color depths increases UPF values. For example, in the case of the madder dyed samples when the K/S value increased from 0.20 to 0.38 the UPF values rose from 11.1 to 16.6. However, the relationship of K/S with UPF is limited to the same fabric type and the results cannot be generalized across fabrics of different weave structures. A primary reason for this observation is the acknowledgement that UPF values are dependent on a multitude of fabric construction factors such as pores in the fabric, thickness, and weight in addition to processing parameters such as dyeing and finishing. Another probable reason is the dependence of K/S on the absorbing properties of colorants in the visible region of the spectrum and that may not influence the absorption characteristics of colorants in the UV region.

**Figure 2:**
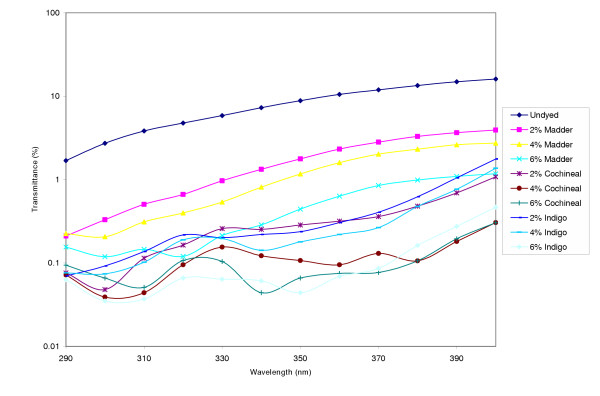
UV transmission of twill weave fabric in the absence and presence of colorants.

The percent UV transmittance data in the presence and absence of colorants for the twill weave fabric is shown in Figure 2. UPF values and protection categories for the dyed twill weave fabric are shown in Tables 3. The twill weave fabric which prior to dyeing was rated as offering Good UV Protection moved to the Excellent UV Protection classification irrespective of the colorant and the concentration of the dye used. Again, it was found that dark colors within the same fabric type transmit less UV radiation than light colors and consequently have higher UPFs.

**Table 3:** UPF values, protection class and K/S values of twill weave fabric dyed with natural colorants at different concentrations

	Colorant	UPF	UV Protection Class	K/S
Twill weave	2% Madder	> 50	Excellent	0.27
	4% Madder	> 50	Excellent	0.44
	6% Madder	> 50	Excellent	0.59
	2 % Cochineal	> 50	Excellent	0.82
	4% Cochineal	> 50	Excellent	1.70
	6% Cochineal	> 50	Excellent	1.89
	2% Indigo	> 50	Excellent	2.33
	4% Indigo	> 50	Excellent	3.76
	6% Indigo	> 50	Excellent	4.00

**Figure 3:**
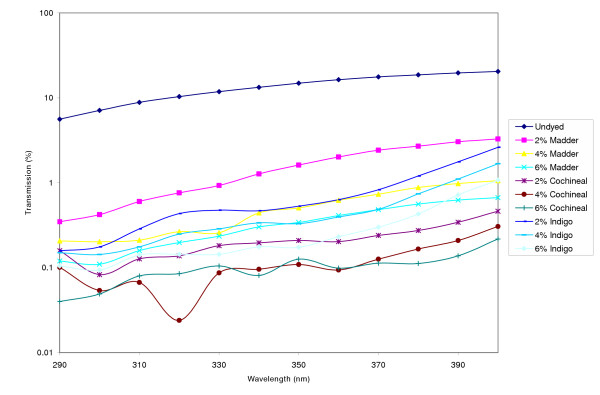
UV transmission of sateen weave fabric in the absence and presence of colorants.

Table 4 shows the UPF values and protection categories for the dyed sateen weave fabric. The percent UV transmittance data in the presence and absence of colorants for the sateen weave fabric is shown in Figure 3. The increase in UPF values of the sateen weave dyed fabrics was dramatic in the sense that the sateen which prior to dyeing could not be rated (UPF < 15) achieved the Excellent UV Protection classification by virtue of its UPF values increasing by more than a factor of four (UPF > 50). This result was true for all colorants and at all dye concentrations. Again, as was the case with the dyed plain weave fabric, the color strength (K/S) of the cochineal and indigo dyed twill and sateen fabrics were higher than the color strength of the madder dyed fabrics conclusively establishing that indigo and cochineal colorants resulted in deeper colors on the fabrics.

**Table 4:** UPF values, protection class and K/S values of sateen weave fabric dyed with natural colorants at different concentrations

	Colorant	UPF	UV Protection Class	K/S
Sateen weave	2% Madder	> 50	Excellent	0.25
	4% Madder	> 50	Excellent	0.36
	6% Madder	> 50	Excellent	0.59
	2% Cochineal	> 50	Excellent	1.78
	4% Cochineal	> 50	Excellent	1.87
	6% Cochineal	> 50	Excellent	2.42
	2% Indigo	> 50	Excellent	1.66
	4%Indigo	> 50	Excellent	2.05
	6% Indigo	> 50	Excellent	2.40

## Conclusions

Fabric weight and thickness are important predictors of UPF values for undyed cotton fabrics. In general, it was found that increase in weight and thickness increased the UPF though the relationship was not linear. UPF of undyed fabrics was significantly enhanced by dyeing with natural colorants especially for fabrics such as the plain weave and the sateen weave fabrics that displayed no protective abilities in the undyed state. The degree of protection imparted after dyeing was a function of the concentration of the colorant in the fabric. Within the same fabric type, as the percentage depth of shade increased so did the UPF values. In addition, darker colors such as indigo provide better protection on account of higher UV absorption. Based on the results of this study it can be theorized that plain, twill or sateen weave cotton fabrics dyed with natural colorants can provide good protection against ultraviolet rays with the only condition being that either the color has to be a dark hue or the concentration of the colorant in the fabric has to be high.

## Competing interests

The author(s) declare that they have no competing interests.

## Authors' contributions

AKS conceived the study, carried out the dyeing and testing and drafted the manuscript.

## Pre-publication history

The pre-publication history for this paper can be accessed here:


